# Epidemiological characteristics and influential factors of hand, foot, and mouth disease reinfection in Wuxi, China, 2008–2016

**DOI:** 10.1186/s12879-018-3385-1

**Published:** 2018-09-19

**Authors:** Chao Shi, Juan Liu, Ping Shi, Hong Ji, Yuan Shen, Yan-Hua Qian

**Affiliations:** 1 Wuxi Center for Disease Control and Prevention, Wuxi, 214023 Jiangsu China; 2 Jiangsu Center for Disease Control and Prevention, Nanjing, 210009 Jiangsu China

**Keywords:** Hand, Foot, And mouth disease, Reinfection, Epidemiology

## Abstract

**Background:**

Hand, foot, and mouth disease (HFMD) is a viral disease caused by human enteroviruses. Although HFMD reinfection is common, studies investigating this phenomenon are insufficient.

**Methods:**

The present study focused on HFMD reinfection in Wuxi from 2008 to 2016 using surveillance system data.

**Results:**

Of 107,677 cases included in the study, 6470 cases were classified as reinfections. The overall reinfection rate was 6.01% (6.37% male and 5.48% female patients), which decreased with increasing age (χ^2^ = 1125.477, *p* < 0.001). The rate was 6.17 and 5.79% in urban and rural areas, respectively, and 7.83 and 5.98% of the cases were severe and mild, respectively. Multivariate logistic regression analysis showed that male sex, younger age, residence in an urban area, and severe disease were risk factors for HFMD reinfection. The case-severity rate in secondary infection cases was lower than that in non-reinfection cases (odds ratio 0.675, 95% confidence interval 0.526–0.866).

**Conclusions:**

Boys younger than 4 years of age living in urban areas were more prone to reinfection. Specific health education and intervention should be developed to protect these susceptible populations.

## Background

Hand, foot, and mouth disease (HFMD) is a common viral disease usually affecting infants and children, but it can also affect adults. It is characterised by fever, mouth ulcers, and vesicles on the hands, feet, or hips [1, 2]. HFMD is a highly contagious disease caused by a group of human enteroviruses; enterovirus 71 (EV71) and coxsackievirus A16 (CoxA16) are considered the primary pathogens [3, 4]. The incubation period of HFMD is 3–7 days, and patients generally recover in 7–10 days [5]. The disease is a mild, self-limiting disorder, and most affected individuals can recover without complications. However, some patients may progress to develop a severe syndrome including myocarditis, neuronal pulmonary oedema, and aseptic meningitis, leading to fulminant cardiorespiratory failure or even death [6, 7].

Epidemics of HFMD have escalated in the Asia-Pacific region since the 1990s, especially in East Asia and Southeast Asia, including Malaysia, Taiwan (China), Singapore, and Mainland China [8–11]. In 1997, 29 patients died in Malaysia, and in 1998, a large epidemic occurred in Taiwan, where a severe form of the disease was reported in 405 patients, 78 of whom died [9, 10]. The largest Asia-Pacific pandemic was reported in China in 2008, when an outbreak of HFMD occurred in Fuyang, north of the Anhui Province, resulting in 22 deaths [12]. Thus, HFMD became an important public health issue in Mainland China and was categorised as a class C notifiable infectious disease by the Ministry of Health of China on 2 May 2008. Since then, medical institutions have been required to report HFMD cases within 24 h.

Owing to a lack of cross-protection among different virus subtypes, HFMD reinfection is quite common [13, 14], which increases the incidence of HFMD and the burden of HFMD on the public health system. However, there are currently insufficient studies on reinfection. The aim of the present study on reinfection in Wuxi was to explore the epidemiological features and factors influencing reinfection.

## Methods

### Data collection

Presently, HFMD is reported as a statutorily notifiable infectious disease, through clinical diagnoses or laboratory-confirmed cases. Data on HFMD cases from 2 May 2008 to 31 December 2016 were extracted from the National Infectious Disease Surveillance System according to the date of onset and the patient’s current address, including name, sex, age, birth date, phone number, name of parents, address, case classification (clinical or laboratory), severity (severe or mild), date of diagnosis, death status, and virus type (EV71, CoxA16, or other enterovirus) for laboratory-confirmed cases.

### Case definitions

The diagnostic criteria of HFMD was based on the Hand Foot and Mouth Disease Clinic Guidelines (2010 edition) issued by the Ministry of Health of China. A clinically diagnosed case was defined as a patient with vesicular rash on hands, feet, mouth, or buttocks, with or without fever, whereas a laboratory-diagnosed case was defined as a clinically diagnosed case with laboratory evidence of enterovirus infection (EV71, CoxA16, or other enterovirus) detected by reverse transcriptase polymerase chain reaction or virus isolation.

Cases were classified as severe, either by clinical or laboratory diagnosis, if the patients presented with any neurological complications, cardiopulmonary complications, or both. Otherwise, they were classified as mild. The reinfection cases were defined as patients who were infected with HFMD at least twice from 2008 to 2016, and the non-reinfection cases were defined as patients who were infected with HFMD only once.

### Reinfected case screening criteria

The screening criteria for reinfected cases included: 1) the patient’s name was the same; 2) > 17 days between the two dates of diagnosis; 3) more than one item common among the birth dates, parent’s name, phone number, and current address. If only one item was the same, the information was checked with the patient’s guardians.

### Statistics

Categorical variables are presented as numbers and percentages, and continuous variables as median and interquartile range (IQR). The chi-square test was used to stratify comparisons of reinfection rate, and the chi-square test for trend was applied to analyse the incidence of infection with respect to age. Independent risk factors of HFMD reinfection were assessed using logistic regression analysis. Variables significant in the univariate analysis were included in a multivariate model. Analyses were performed with SPSS version 11.0 (SPSS, Chicago, IL, USA). All testing was two-sided, and a *p* value < 0.05 was considered statistically significant.

## Results

### General patient information

Of the 107,677 cases of HFMD reported from 2008 to 2016 in Wuxi, 6470 cases were identified as reinfections. The reinfection rate was 6.01%, with 6109 patients infected twice (5.67%), 346 patients infected three times (0.32%), 14 patients infected four times (0.0013%), and one patient infected five times (0.0001%). Of all the non-reinfection HFMD cases, 1635 were classified as severe, with five deaths. On the other hand, 128 of the reinfection HFMD cases were severe, but no deaths were reported.

The reinfection rates in different groups are presented in Table 1. The reinfection rates in male and female patients were 6.37 and 5.48%, respectively. The reinfection rate in children aged under 1 year of age was 9.65% and decreased with increasing age (χ^2^ = 1125.477, *p* < 0.001). The reinfection rate in children younger than 4 years of age was significantly higher than that in children over 4 years of age, with children under 4 years of age accounting for 86.82% of primary infections. For the different status, the reinfection rate was highest in the scattered children (7.24%). The reinfection rate was higher in urban areas (6.17%) than in rural areas (5.79%). The reinfection rate was 7.83% for the severe cases and 5.98% for the mild cases.

**Table 1:** Repeated infection rate of HFMD population of different features in Wuxi city

Characteristics	Case (n)	Reinfection (n)	Non-Reinfection (n)	Refection rate (%)	χ^2^	*P*
Sex					36.686	< 0.001
Male	63,671	4058	59,613	6.37		
Female	44,006	2412	41,594	5.48		
Age (year)					1138.277	< 0.001
0	7087	684	6403	9.65		
1	28,202	2397	25,805	8.50		
2	20,745	1377	19,368	6.64		
3	20,814	1159	19,655	5.57		
4	15,134	589	14,545	3.89		
≥ 5	15,695	264	15,431	1.65		
status					572.368	< 0.001
Scattered children	67,122	4857	62,265	7.24		
Kindergartens children	36,024	1581	34,443	4.39		
School students	4149	32	4117	0.77		
Others	382	0	382	0		
Residence					6.729	0.009
Rural	45,397	2627	42,770	5.79		
Urban	62,280	3843	58,437	6.17		
Clinical classification					9.738	0.002
Mild	106,042	6342	99,700	5.98		
Severe	1635	128	1507	7.83		

### Seasonal distribution and time interval of reinfection

**Fig. 1:**
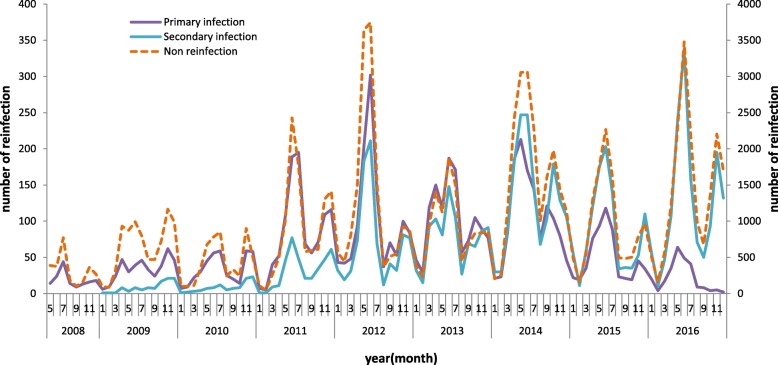
Seasonal distributions of the primary and secondary infections and non-reinfection

The seasonal distributions of the primary infection, secondary infection, and non-reinfection cases were similar. There were two peaks, which occurred in the seasonal months of May to July and November to December annually (Fig. 1).

**Fig. 2:**
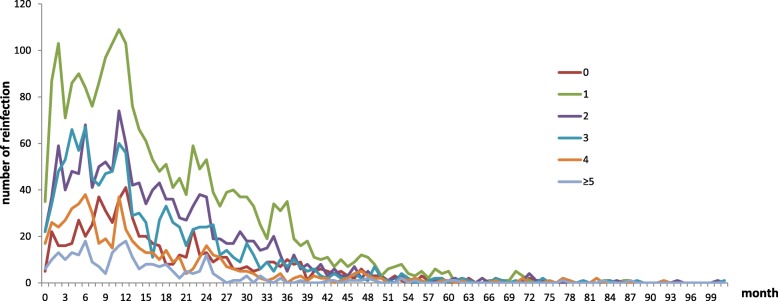
Time intervals between primary and secondary infections in different age groups

In patients who were infected twice, the median time interval between the two infections was 13 (IQR 7–24) months. In patients who were infected three times, the median time intervals were 10 (IQR 5–17) and 11 (IQR 5.75–20) months between the previous two infections and the latter two infections, respectively. In patients who were infected four times, the median time intervals were 9.5 (IQR 8.5–14.5), 7.5 (IQR 3–11.75) and 11 (IQR 4–19.25) months, respectively. In patients who were infected five times, the median time intervals of reinfection were 5, 16, 2, and 12 months respectively. For the different age groups (0, 1, 2, 3, 4, and 5 years), the proportions of cases in which the time interval between the primary and secondary infections was within 2 years were 74.12, 73.84, 78.65, 80.93, 81.32, and 87.12%, respectively (Fig. 2).

### Relevant factors and factors influencing reinfection

Sex, age, status, residence, and clinical classification were relevant factors for HFMD reinfection (Table 1). From the multivariate regression analysis, sex, age, and residence were found to have a significant influence on reinfection (*p* < 0.05), with male sex, younger age, and living in an urban area being risk factors of HFMD reinfection. The detailed results are listed in Table 2.

**Table 2:** Multivariate Logistic regression analysis of influencing factors of HFMD reinfection in Wuxi city

Factors	β	wald	OR (95% *CI*)	*P*
Sex				
Female			1.000	
Male	0.158	35.114	1.171 (1.111–1.234)	< 0.001
Age (year)				
0	1.721	405.358	5.591 (4.728–6.610)	< 0.001
1	1.582	408.741	4.865 (4.173–5.672)	< 0.001
2	1.298	276.805	3.662 (3.142–4.267)	< 0.001
3	1.054	205.244	2.869 (2.484–3.314)	< 0.001
4	0.649	68.821	1.915 (1.642–2.232)	< 0.001
≥ 5			1.000	
Residence				
Urban	0.111	17.663	1.117 (1.061–1.176)	< 0.001
Rural			1.000	
Clinical classification				
Mild			1.000	
Severe	0.189	3.969	1.208 (1.003–1.455)	0.05

### Clinical classification and virus subtype of reinfection

Of all the reinfection cases, 128 were classified as severe. Among these, 65 severe cases occurred during the second infection. The case-severity rate in the primary infection cases (1.98%) was higher than that in both the second infection cases (1.01%) [odds ratio (OR) 1.969, 95% confidence interval (CI) 1.458–2.660), and non-reinfection cases (1.49%) (OR 1.329, 95% CI 1.107–1.594); however, the case-severity rate in secondary infection cases was lower than that of the non-reinfection cases (OR 0.675, 95% CI 0.526–0.866) (Table 3).

**Table 3:** The case-severity rate in different groups

Groups	Severe (n)	Mild (n)	Case-severe rate (%)	OR (95%CI)	*χ* ^*2*^	*p*
Reinfection cases				1.969 (1.458–2.660)	20.263	< 0.001^a^
Primary infection	128	6342	1.98	1.329 (1.107–1.594)	9.406	0.002^b^
Second infection	65	6405	1.01	0.675 (0.526–0.866)	9.674	0.002^c^
Non-reinfection cases	1507	99,700	1.49	1.000		

^a^The P value was primary infection group compares with second infection group

^b^The *P* value was primary infection group compares with non-reinfection group

^c^The *P* value was second infection group compares with non-reinfection group

Of all the reinfection cases, 478 were laboratory-diagnosed cases, of which 229 were tested during only the primary infection, 240 cases during only the second infection, and 19 cases during both infections. The proportion of patients infected with CoxA16 in the primary infection cases (25.76%) was lower than that in the non-reinfection cases (36.16%) (OR 0.613, 95% CI 0.452–0.830). However, the proportion of patients infected with other enteroviruses in the primary infection cases (25.76%) was higher than that in the non-reinfection cases (16.95%) (OR 1.701, 95% CI 1.249–2.315) (Table 4).

**Table 4:** The objects pathogenic distribution in different groups

Groups	Laboratory diagnosed	Virus subtypes								
		EV71			CoxA16			Other		
		n (%)	*P*	OR (95%CI)	n (%)	*P*	OR (95%CI)	n (%)	*P*	OR (95%CI)
Reinfection cases			0.567^a^	1.112 (0.773–1.598)		0.073^a^	0.694 (0.465–1.035)		0.206^a^	1.319 (0.858–2.027)
Primary infection	229	111 (48.47)	0.641^b^	1.066 (0.816–1.392)	59 (25.76)	0.001^b^	0.613 (0.452–0.830)	59 (25.76)	0.122^b^	1.289 (0.933–1.781)
Second infection	240	110 (45.83)	0.751^c^	0.958 (0.738–1.246)	80 (33.33)	0.376^c^	0.883 (0.669–1.164)	50 (20.83)	0.001^c^	1.701 (1.249–2.315)
Non-reinfection cases	3711	1740 (46.89)		1.000	1342 (36.16)		1.000	629 (16.95)		1.000

^a^The P value was primary infection group compares with second infection group

^b^The P value was primary infection group compares with non-reinfection group

^c^The P value was second infection group compares with non-reinfection group

Of the 19 patients who had both primary and secondary laboratory test results (Table 5), two were infected with EV71 and three with CoxA16 in both infections. Both patients who were infected with EV71 were aged 1 year, with infections occurring in April and July 2015 and in June 2010 and January 2013, respectively. Of three patients who were infected with CoxA16, the first was a 2-year-old boy, with infections occurring in August 2013 and April 2014. The second and third were 4-year-old-boys, with the infections occurring in March 2015 and June 2015 and in September 2015 and August 2016, respectively.

**Table 5:** Pathogenic results in subjects whose pathogens were detected in two infections

Primary infection	Secondary infection	Number
EV71	EV71	2
EV71	COXA16	5
EV71	Other enteroviruses	1
COXA16	EV71	4
COXA16	COXA16	3
Other enteroviruses	EV71	2
Other enteroviruses	COXA16	2

## Discussion

The HFMD reinfection rate was 6.01% in Wuxi during 2008 to 2016, which is higher than that in Anhui Province from 2008 to 2013 [14]. Regional differences and a longer investigation period in Wuxi may have accounted for the discrepancy. However, our results are similar to a study in Fujian Province [15]. Most of the reinfection cases were patients who were infected twice (94.42%), with one patient being infected five times in 3 years. Our results also demonstrated that the HFMD reinfection rate in boys was higher than that in girls, which is in accordance with the incidence of HFMD [16]. This may be attributed to the more active lifestyle of boys compared with girls, making them prone to touching objects polluted by infected children [14].

The present study revealed the reinfection rate decreased with increasing age, and the reinfection rate in children younger than 4 years was significantly higher than that in children over 4 years, with children under 4 years of age accounting for 86.82% of primary infection cases. Ji et al. [17] indicated that the seroprevalence rate of anti-EV71 and anti-CoxA16 gradually increased with age and reached a peak in 4-year-olds. Moreover, we found the median time interval between reinfection was 13 (IQR 7–24) months, and therefore, children under 4 years of age were more prone to reinfection within 1–2 years after the first HFMD infection.

Descriptive analysis revealed that HFMD reinfection mainly existed in scattered and kindergarten children, with the reinfection rate in the scattered children being higher than that in the other statuses. One possible reason was that the scattered children were young and had not yet developed proper personal hygiene, suggesting the need for families, and particularly caretakers, to pay attention to the personal and environmental hygiene of children. In addition, we observed that the reinfection rate was higher in urban areas, which may be attributed to the high population density and increased floating population in these urban areas.

Multivariate analysis results indicated that male sex, younger age, living in an urban area, and severe disease were risk factors of HFMD reinfection. According to the results, boys younger than 4 years, living in urban areas, and classified as having a severe first infection may be deemed a population very susceptible to reinfection. Therefore, parents or guardians need to pay close attention to the signs of HFMD. In addition, administrations need to develop targeted health education for susceptible populations of reinfection. The seasonal distributions of primary infection, secondary infection, and non-reinfection cases were similar. Our results demonstrated that during HFMD prevalence, children, especially the population susceptible to reinfection, should be monitored, even if they have been previously infected.

The case-severity rate of the primary infection was highest for different infection statuses. However, the case-severity rate of the second infection was lower than that of non-infection (OR 0.675,95% CI 0.526–0.866). This trend may be attributed to the increased age of patients during the second reinfection or possibly a suboptimal acquired immune protection from the initial primary infection.

The proportion of patients infected with CoxA16 in primary infection cases was lower than that in non-reinfection cases (OR 0.613, 95% CI 0.452–0.830). However, the proportion of patients infected with other enteroviruses in primary infection cases was higher than that in non-reinfection cases (OR 1.701, 95% CI 1.249–2.315). This showed that the patients infected with CoxA16 were less prone to reinfection, although other enteroviruses were more likely to cause reinfection.

Two studies [14, 18] both found two HFMD patients infected twice with EV71. Xie et al. [19] also reported one patient infected with EV71 twice and one patient infected with Cox A16 twice. In the present study, two patients were infected with EV71 twice, and three patients were infected with Cox A16 twice. EV71 and Cox A16 have several genogroups [20–22], and more than one genogroup of EV71 or Cox A16 virus can occur simultaneously in an epidemic. Based on molecular typing, EV71 has been classified into three genotypes (A, B, and C) based on a partial VP1 sequence analysis [6], and in patients infected with genogroup B or C EV71 virus, cross-protection against genogroup A is not guaranteed [23]. Therefore, cross-infection among patients with the different genogroups of EV71 or Cox A16 virus is possible.

In the present study, 2–5% of patients underwent laboratory testing; therefore, laboratory results for both infections were available for only a few reinfection cases. Moreover, the samples of two patients infected with the same virus subtype in both infections could not be collected for sequence analysis. Further research emphasising key strata theory is warranted.

## Conclusion

This study indicated that the reinfection rate of HMFD in Wuxi from 2008 to 2016 was 6.01%. The population susceptible to HFMD reinfection was boys younger than 4 years of age who live in urban areas. It is important that administrations develop targeted health education and interventions to reduce the reinfection rate in susceptible populations.

## Abbreviations

CI: Confidence interval
CoxA16: Coxsackievirus A16
EV71: Enterovirus 71
HFMD: Hand, foot, and mouth disease
IQR: Interquartile range
OR: Odds ratio
